# The Sexual Dimorphism of Human Adipose Depots

**DOI:** 10.3390/biomedicines10102615

**Published:** 2022-10-18

**Authors:** Nathalie Boulet, Anais Briot, Jean Galitzky, Anne Bouloumié

**Affiliations:** Inserm, Unité Mixte de Recherche (UMR) 1297, Team 1, Institut des Maladies Métaboliques et Cardiovasculaires (I2MC), Université de Toulouse, F-31432 Toulouse, France

**Keywords:** obesity, gluteofemoral, brown adipose tissue

## Abstract

The amount and the distribution of body fat exhibit trajectories that are sex- and human species-specific and both are determinants for health. The enhanced accumulation of fat in the truncal part of the body as a risk factor for cardiovascular and metabolic diseases is well supported by epidemiological studies. In addition, a possible independent protective role of the gluteofemoral fat compartment and of the brown adipose tissue is emerging. The present narrative review summarizes the current knowledge on sexual dimorphism in fat depot amount and repartition and consequences on cardiometabolic and reproductive health. The drivers of the sex differences and fat depot repartition, considered to be the results of complex interactions between sex determination pathways determined by the sex chromosome composition, genetic variability, sex hormones and the environment, are discussed. Finally, the inter- and intra-depot heterogeneity in adipocytes and progenitors, emphasized recently by unbiased large-scale approaches, is highlighted.

## 1. Introduction

The two main types of adipose tissues (AT), the white (WAT) and the brown (BAT), are distributed throughout the body in several depots which exhibit different metabolic features. WAT is localized in subcutaneous (abdominal and gluteofemoral) and visceral depots, while BAT is present in internal locations in the majority around the shoulders and neck [1]. The distribution of body fat, which differs according to sex, is now well recognized as a major determinant of health. Indeed, the accumulation of fat in the truncal part of the body and particularly in visceral AT (VAT) increases the risk to develop cardiometabolic disease and even all-cause mortality, while the gluteofemoral depot is protective [2,3]. Conversely, the presence of BAT has been associated with a lower prevalence of cardiovascular and metabolic diseases [1]. Importantly, the responses to the obesogenic environment and energy-restricted conditions and their cardiometabolic outcomes are not equivalent in men and women [4,5,6] and nowadays, in most countries, the prevalence of obesity is higher in adult women than in men [7,8]. The sex-related differences in socioeconomic factors including educational attainment and occupational status are key obesity drivers [9]. In addition, gender defined by the World Health Organization (WHO) as “the socially constructed characteristics of women and men—such as norms, roles and relationships of and between groups of women and men” certainly influences the behavioral determinants of body fat and its distribution [10]. The present review will be limited to the differences related to sex as defined by the WHO as “the different biological and physiological characteristics of males and females, such as reproductive organs, chromosomes, hormones, etc.”. Few comparative studies of diet-induced obesity with the commonly used rodent models including mice and rats highlight marked sex-divergent responses depending on the species in terms of food preference and intake, adiposity, fat depot repartition, locomotor activity and BAT activation [11,12]. This review aims to summarize the current knowledge on sexual dimorphism focusing on human data when available since sexual dimorphism in adiposity and fat depot repartition is a specific feature of the human species [13]. The sexual dimorphism of fat depots during a lifetime and its consequences on health as well as the drivers of the sex differences will be discussed.

## 2. Sex Differences in Fat Depots in Lifespan and Aging

**Figure 1:**
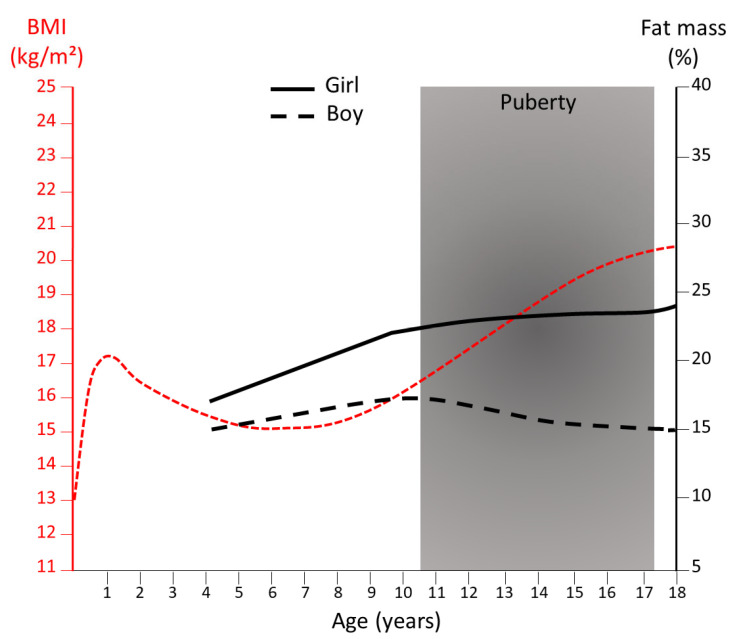
Evolution of body mass index and fat mass percentage from childhood to early adulthood. The body mass index (BMI) curve (in red) is extracted from the 50th percentile in boys and girls from IOTF (International Obesity Task Force) and the % body fat from the 50th percentiles of body fat reference curves for boys (dotted black line) and girls (solid black line) [19].

The majority of mammals have a very low-fat mass at birth, with the noticeable exception of the human species [14] characterized by the fetal development of BAT and WAT, starting at the mid-end of the second trimester of gestation [15]. The human newborn further gain adiposity until 6 months of age followed by a reduction in the fat mass during infancy [16]. At puberty onset, the expansion of adiposity becomes sex-dependent with girls experiencing a rapid increase in total fat mass [16] (Figure 1). From late puberty to early adulthood [17] women exhibit approximately 10% higher body fat mass compared with men for the same body mass index (BMI) [18] (Figure 1). With aging, in both sexes, a progressive increase in whole-body adiposity is generally observed with the maintenance of higher adiposity in women compared with men.

**Figure 2:**
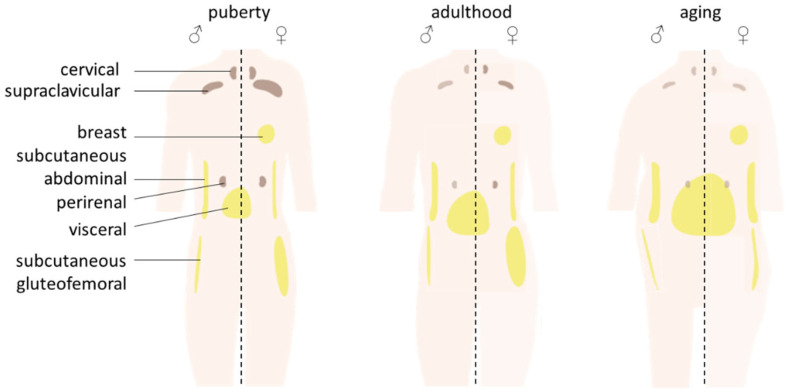
Evolution of the distribution of white and brown fat depots in men and women.Distribution of WAT (yellow) and BAT (brown) fat depots in men (**left**) and women (**right**) at puberty, adulthood and aging. Already at puberty, men exhibit more truncal fat (visceral and subcutaneous abdominal AT) while women have more subcutaneous gluteofemoral fat and supraclavicular BAT. During adulthood, the differences in WAT distribution are exacerbated. With aging in both men and women, the ratio of visceral over subcutaneous AT increases. BAT depots decrease all life long, with a higher impact in men than women.

The distribution of BAT and WAT fat depots follows sex- and age-dependent trajectories (Figure 2). Sexual dimorphism in WAT depot repartition is apparent even prepubertally with girls having less waist fat and more hip fat than boys [20]. The magnitude of the sex difference is amplified with maturation. Women tend to accumulate fat in the lower part of the body (gluteofemoral subcutaneous AT gfSAT) while men in the central truncal part (abdominal SAT and visceral AT VAT) [20,21]. The development of the breast at puberty is mainly related to the increase in the amount of fibrous and adipose tissues. With age, in both men and women, the ratio of VAT over SAT increases with the accumulation of VAT [21]. Aging is also associated with an accumulation of bone marrow AT [22] with a reversion of sex differences and discrete age- and gender-specificity according to bone location [23].

All along infancy, the cervical-supraclavicular region constitutes the major BAT depot [24] with the presence of brown-like adipocytes in perirenal and visceral depots as well as within subcutaneous inguinal WAT [25]. The activation of the supraclavicular BAT is higher in girls [26] as early as 12 months old [27]. BAT volume and activity increase at puberty regardless of sex [24]. In adults, data are controversial concerning the increased BAT prevalence, volume and activation in women compared with men [28,29,30,31]. In both sexes, BAT amount and activity are decreased with aging [28] with a stronger impact in men [32].

## 3. Sex Differences in Fat Depots and Reproductive Health

From an evolutionary biology perspective, the sexual dimorphism of WAT repartition in the human species is thought to be a trait related to reproductive function. In women, puberty, pregnancy and menopause are associated with changes in fat mass repartition [33,34]. The initial phase of gestation is associated with a gradual increase in maternal fat stores until the end of the second trimester with a preferential accumulation in the VAT compartment [35]. The mammary AT is progressively replaced by alveoli [36]. Menopause is also associated with an increase in VAT and a concomitant decrease in gfSAT [37]. The mammary gland is gradually replaced by AT and fat accumulates in the bone marrow.

The gfSAT has long been considered as the energy reserve specifically mobilized during lactation [38]. The lipostat hypothesis in reproduction underlies that a critical level of fat in women is necessary for menarche and for the recovery of menstrual cycles after energy restriction [39]. Leptin, the adipokine secreted in proportion to fat mass, is thought to be the molecular basis of the lipostat by acting on the hypothalamic-pituitary-gonadal axis [40]. It is expressed in women in higher quantities in the gfSAT than in the abdominal depot [41]. Obesity has been identified as a risk factor for subfertility in women, especially with ovulation disorders [42] and outcomes of infertility treatment [43]. In addition to the total amount of body fat, the adequate storage and availability of metabolic substrates are more probably decisive for optimal reproductive function [3,44] as well as for maternal-fetal metabolic communication during pregnancy [45]. In agreement, waist circumference in women is inversely related to the probability of live birth among women undergoing assisted reproductive technology independently of BMI [46]. Although fewer data are available, male fertility is also impacted by adiposity and fat depot repartition since negative associations have been reported between waist circumference and semen parameters [47]. The link between fertility and fat depots may involve hormonal changes including leptin but also peripheral conversion of sexual steroids in fat depots. Aromatase converts androstenedione to estrone and testosterone into estradiol. It exhibits fat depot- and age-dependent expression with the highest levels in the buttocks, followed by the thighs, and lowest in the abdomen [48,49] and males show higher basal aromatase activity than females whatever the fat depots [49].

## 4. Sex Differences in Fat Depots and Cardiometabolic Health

Epidemiologic studies have consistently demonstrated a link between adiposity (estimated by BMI) and cardiometabolic health, including insulin resistance, T2D (type 2 diabetes), CVD (cardiovascular diseases) but also with cancers [50].

The association of higher adiposity and cardiometabolic risks begins earlier in the life course in men than in women and is stronger until midlife, particularly for atherogenic lipids [51]. The inverse is true when considering partial lipodystrophies in which the metabolic abnormalities manifest earlier and are more severe in women [52]. The enhanced incidence of some cancers with obesity also exhibits sex differences with men having a stronger association between BMI and hepatocellular carcinoma or colorectal cancer risks than women [53]. Moreover, high BMI is more strongly associated with all causes and cardiovascular mortality in men than in women [50].

Independently of BMI, the fat depot distribution, assessed either by the waist circumference (WC) or by the waist-to-hip ratio (WHR) adjusted or not to BMI (WHRadjBMI) is also a strong determinant of health and the threshold values of both WC and WHR for cardiometabolic health do take into account the sexual differences (Table 1).

Increased visceral AT and decreased femoral fat are now recognized as variables defining metabolically unhealthy individuals, even in absence of obesity [54]. The association of risk factors and WHR is also dependent on sex, for example when considering fasting insulin [55] and the risk of myocardial infarction [56]. Finally, recent data highlight the protective impact of BAT against T2D, dyslipidemia, cardiovascular pathologies and hypertension and this protection is also observed in overweight or obese patients [1].

The respective contribution of each individual fat depot in health remains to be fully established. Greater WHRadjBMI can be causally linked to the risk of cardio-metabolic diseases through either relatively lower gfSAT or higher VAT or both [57]. The association between WC and excess risk of mortality regardless of the BMI [58] strongly suggest that the accumulation of fat in the abdominal cavity may be by itself causal of cardio-metabolic disorders. In addition, a possible independent protective role of the gfSAT is emerging [3,59]. A recent study demonstrated that distinct sets of genetic variants associated with a higher WHR but either with lower gfSAT or with higher VAT are both associated with higher risks of T2D and CVD [60]. Inversely, the stratification of individuals based on the polygenic scores relevant for the volumes of gfSAT, abdominal SAT (aSAT) or VAT showed that the individuals with higher gfSAT scores exhibited better cardio-metabolic profile with higher HDL-cholesterol, lower plasma triglycerides and lower risks of T2D and CVD [61]. Therefore, the inability of gfSAT to expand may be a determinant in unhealthy fat distribution promoting central fat depots. Interestingly, a prospective randomized controlled trial in non-obese women showed that following suction lipectomy, body fat was redistributed from the thigh to the abdomen, suggesting that lack of gfSAT is counterbalanced by the development of aSAT [62]. Concerning human BAT, few studies are available. The presence of active BAT is associated with favorable fat depot distribution and improved metabolic health, independently of sex [63]. An inverse correlation between cold-activated supraclavicular BAT and VAT amount has also been shown [64]. However, whether all the brown fat depots contribute similarly to cardiometabolic protection remains to be determined.

## 5. Determinants of the Fat Depot Repartition According to the Sex

The drivers of sex differences result from complex interactions between the sex determination pathways determined by the combination of sex chromosomes, genetic variability, sex hormones and environment.

### 5.1. Genetic Determinants

At fertilization, the combination of the sex chromosomes establishes the biological sex, XX for females and XY for males. About 900 genes are expressed on the X chromosome while about 55 genes are on the Y chromosome. Following the differentiation of gonads, it is challenging to discriminate the effects related to sex chromosomes from the ones due to sex steroid hormones. The most widely used model to distinguish gonadal and chromosome sex effects is known as the Four Core Genotypes (FCG) mouse model with mice having either XX or XY chromosomes on both male and female gonadal backgrounds. XX mice, regardless of having ovaries or testes, exhibit a higher proportion of fat mass highlighting the major role of X chromosomes in regulating adiposity [65].

Concerning genes carried by autosomal chromosomes, the studies of extreme forms of early onset obesity in humans identified genes with major influence on the central nervous system in the control of adiposity including the leptin-melanocortin pathway (*LEP*, *LEPR*, *POMC*, *PCSK1*, *MC4R*) [66]. Studies of monogenic lipodystrophy syndromes highlighted the contribution of single genetic variants in fat depot distribution with roles in AT biology such as adipocyte differentiation (PPARG) or lipid droplet function (*PLIN1*, *BSCL2*, *CAV1*, *CAVIN1*, *CIDEC*) [67]. Meta-analysis of large-scale single-nucleotide polymorphism-based genome-wide association studies (GWAS) for BMI, WC and WHRadjBMI in whole population based-biobanks demonstrated the polygenic contributions of multiple loci, each taken individually having small effects in adiposity and fat mass repartition [68,69]. The strongest GWAS signal for BMI is the *FTO* locus, for WC the *MC4R* locus and for WHRadjBMI the *RSPO3* locus [69]. Expression quantitative trait loci (eQTLs) analysis in relevant tissues together with exome sequencing further highlighted enrichment in the brain- or peripheral-tissue-related pathways as a determinant for BMI or WHRadjBMI, respectively. For example, the predictive loss of function (pLoF) of adipocyte-expressed *PLIN1*, *INSR*, *ACVR1C* and *PDE3B* and liver-expressed *INHBE* variants are associated with increased gfSAT and healthy metabolic phenotypes [61,70,71]. Importantly, WHRadjBMI-associated loci exhibit heritability and effect size stronger in women than men with one-third of all signals sexually dimorphic [72].

### 5.2. Epigenetic Mechanisms

In addition to genetic variation, epigenetic mechanisms contribute to the sex dimorphism in adiposity and fat mass repartition. Exposure to harmful factors (malnutrition, pollutants, stress, endocrine disruptors) during pregnancy reprograms the expression of certain genes altering adiposity in adults and with different responses between women and men [73]. Female offspring of a mother suffering undernutrition during pregnancy, as observed during the Dutch famine of 1944–1945, are prone to gain adiposity during adulthood [74], while maternal obesity is more likely to increase adiposity in the male offspring [75]. Epigenetic reprogramming could be transmitted to subsequent generations in the absence of additional exposure via epigenetic modifications of paternal gametes [76].

The two well-described epigenetic mechanisms that primarily contribute to sex differences and arise early in embryo development are genomic imprinting and X chromosome inactivation in females.

Genomic imprinting is an epigenetic regulatory mechanism consisting of the monoallelic expression in the function of the parental origin of a subset of genes in specific regions of the genome. Some imprinting disorders are associated with impaired metabolism and obesity such as the Prader–Willi syndrome characterized by the absence of an expressed paternal copy of the *SNORD116* locus [77]. Another example is the *KLF14* maternal imprinted locus. *KLF14* T2D risk-allele carriers shift body fat repartition from a gynoid to abdominal stores and display a marked increase in adipocyte cell size but in women only [78]. In mice, a recent study demonstrates that maternal obesity impairs the thermogenesis and energy expenditure of BAT, predisposing female offspring to obesity and metabolic dysfunctions via *Dio3os*, a maternally imprinted long-noncoding RNA [79]. It has also been shown that maternal high-fat diet feeding before and during pregnancy affects gene expression in WAT and BAT of the offspring in a sex- and adipose-depot-dependent manner, which may prevent metabolic complications in females but not in males [80].

The process of X chromosome inactivation is complex and leads to a mosaic pattern of cells with the paternal or maternal X active in women. In addition, it may be unbalanced with incomplete gene silencing. A recent study based on gene expression datasets from 44 human tissues showed that gfSAT is the fourth tissue with the largest proportion of sex-differentially expressed genes (VAT ranked at the 11th position) [81]. Overall, the larger sex effects are associated with X-linked genes with higher expression in females (female-biased genes) most of them being potential candidates for escape from X-chromosome inactivation [81].

### 5.3. Cell Determinants

The GWAS highlight several peripheral tissue- and cell-enriched pathways as determinants for WHRadjBMI suggesting that WAT intrinsic cell composition, function and remodeling are key contributors in fat depot repartition [61,70,71]. Recent single-cell atlas of human and mouse WAT provides a transcriptional basis of the heterogeneity in subsets of resident progenitors and adipocytes with fat depot-specific prevalence and functions [82]. A recent study identified 162 sex-biased genes in SAT that were implicated in oxidative phosphorylation and adipogenesis [83] further supporting a role for adipogenesis and adipocyte metabolism in the SAT-related sex differences.

#### 5.3.1. Resident Progenitors

Lineage tracing data in mice revealed the heterogeneity of the embryonic origins of the adipocyte lineages between fat depots and also within fat depots [84]. In addition, sex differences have been reported with male and female adipocytes of the perigonadal AT originating from different mesodermal subcompartments [84]. In humans, a regional expression of developmental and patterning genes is also in favor of distinct origins of fat depots [85]. In addition, single-cell RNA sequencing and flow cytometry datasets provide clear evidence of marked inter- and intra-depot heterogeneity of the resident progenitors [82]. In lean women, gfSAT contains the highest proportion of the bipotent white and beige preadipocytes [86] while greater numbers of promyofibrogenic progenitors are found in VAT compared with SAT of obese women [87]. The higher intrinsic adipogenic potential of gfSAT compared with aSAT has been well established by clinical studies [88] including overfeeding approaches demonstrated that aSAT growth occurs through adipocyte hypertrophy while gfSAT growth is mediated by adipocyte hyperplasia [89]. Thus, gfSAT is considered to have the unique capacity to expand in a healthy manner protecting the other organs from lipotoxicity. Interestingly, in diet-induced obese mice, de novo adipogenesis is observed in both inguinal (SAT) and gonadal (VAT) fat in females but only in VAT in males [90]. The sex differences in inguinal progenitor differentiation have been associated with sex-specific phosphorylation of PPARγ, the master transcription factor involved in adipogenesis [91].

Besides adipogenesis, adipocytes may arise from trans-differentiation, a process by which mature adipocytes may undergo changes from white to brown/beige phenotype and vice versa [92]. In addition, during pregnancy and lactation, the adipocytes from female mice SAT may transdifferentiate into epithelial glandular cells, the so-called “pink adipocytes” [93]. Mesothelial cells may also be a source of adipocytes in VAT from mice [94] and humans [87] although controversial data have been reported [95]. A recent study using integrative correlation analysis of human AT RNA-seq data identified male-only cell-type-enriched transcripts from Y chromosome in VAT adipocytes, progenitors and mesothelial cells [96]. It is therefore tempting to speculate that sex differences in the origins of adipocytes may contribute to the sexual dimorphism in fat depots. However, additional data are required to fully characterize such differences.

#### 5.3.2. Mature Adipocytes

Differences in adipocyte metabolism and more specifically in lipolysis and lipogenesis, contribute to the inter-depot differences in the size of the adipocytes with a decreased gradient from gf SAT to VAT [2]. In the light of recent single-cell atlas [82], other functions will be worthwhile to investigate such as thermogenesis. Regional variations in lipolytic responsiveness due to an altered balance between the lipolytic and antilipolytic receptors have been reported [97]. Taking into account the mean mass of the depots, aSAT is estimated to be the source of 60% of circulating free fatty acids (FFA), gfSAT 15–20% and VAT 6–17% [98]. The importance of lipolysis is underlined by the fact that the catecholamine-activated lipolysis in VAT but not SAT adipocytes, is associated with cardiovascular risk factors with obesity [99]. Although differences according to sex are controversial concerning SAT [100,101,102], the catecholamine-induced rate of FFA mobilization from VAT to the portal venous system is higher in men than in women [103]. Concerning lipogenesis, the aSAT takes up meal fat more efficiently than gfSAT in both women and men [104]. It is associated with higher lipoprotein lipase activity in the vascular lumen, responsible for the hydrolysis of circulating triglyceride-rich lipoproteins [104]. In response to a fat-enriched meal, the efficiency of meal fat uptake into aSAT is similar in both sexes, but women exhibit a greater gfSAT uptake than men [105] with a preference for extracting FA directly from the plasma pool of non-esterified FA and very low-density lipoprotein-triglycerides (VLDL-TG) while FA from chylomicron-TG are preferentially extracted by aSAT [106]. Taken together, FA turnover shows a regional gradient, lower in gfSAT, intermediate in aSAT and highest in VAT. The delivery of FFA from VAT to the liver through the portal system is part of a vicious circle by which central obesity may trigger metabolic dysfunction promoting insulin resistance and resulting in the failure of insulin to further suppress lipolysis [2].

### 5.4. Hormonal Determinants

The majority of sex-biased gene expressions are autosomal, suggesting an influence of sex on genome-wide regulatory programs including epigenetic modulations and/or regulation of promotor activity [81]. Enrichment in transcription factor-binding sites for sex hormone receptors including estrogen (*ESR1*) and androgen (*AR*) receptors and transcription factors (TF) that co-localize with steroid receptors are found in sex-biased gene promotor region in gfSAT, with AR belonging to the top 5 TF [81]. *TBX15*, a transcription factor trans-regulating a network of 347 genes including *PARG*, *KLF15*, *PPARA*, and *ADIPOQ* that control WHRadjBMI and T2D in a sex-dependent manner exhibit an AR response element in its promoter region [107]. TBX15 is of particular interest since it has been involved in the control of brown adipogenesis and identified in a genomic region specifically selected in the Greenland Inuit population and related to the Denisovan genome [108].

Inverse associations of WHR and body mass index (BMI) with total estradiol but positive associations with free estradiol in pre-menopausal women, as well as positive associations with total and free testosterone and inverse associations with sex hormone binding globulin (SHBG) in all women have been reported for the UK Biobank cohort [109]. In men, waist circumference, WHR and BMI were associated inversely with SHBG, total and free testosterone [110]. The pioneering work of Bjorntorp [111] highlights the complex cross-talk between cortisol and insulin (promoting fat storage) and sex steroids (favoring fat mobilization). Indeed, both cortisol and insulin increase lipoprotein lipase activity and adipogenesis. In addition, insulin inhibits lipolysis. The higher glucocorticoid responsiveness of VAT compared with SAT is in favor of central obesity [112]. Conversely, active androgens and estrogens inhibit adipogenesis while stimulating lipolysis [112]. However, the net effects are more complex than first described. Central obesity is associated with low testosterone levels in men and high testosterone levels in women [113]. The age, menopausal status and the presence of other hormonal alterations such as insulin resistance in polycystic ovarian syndrome are certainly major additional factors to take into account in women [113]. To note, the effects of estrogens and progesterone on adipocytes or progenitors are weak in comparison with in vivo effects suggesting that their predominant effects are central [114,115,116].

## 6. Conclusions

Increasing lines of evidence highlight the complex interactions of genetic and epigenetic modifications with sex chromosomes, hormones and cell subsets in the determination of fat depots distribution and function in the human species. The sex dimorphism of the effects and heritability of autosomal coding genes are not fully understood. Early regulatory dialogues between sex and autosome chromosomes are certainly at play but it is worth noting that the X and Y chromosome are often not included in GWAS. In-depth investigations are thus required to fully understand their relative contributions. In addition, the sex-differential representation of specific phenotypes in whole population-based biobanks may constitute a bias to be taken into account [117]. Although some genetic components are highlighted, the mechanisms and molecular actors involved in the protection provided by BAT and gfSAT remain to be fully defined. Increasing lines of evidence highlight the large heterogeneity of adipocytes and progenitor subsets between fat depots but also within the same AT. Whether such heterogeneity contributes to the sex differences in metabolic and/or endocrine AT functions will require additional investigation. Interestingly, large-scale metabolomics and proteomic analyses identified plasma metabolites and proteins associated with WC or WHR independently of their association with BMI [118,119]. An in-depth analysis taking into account potential sex differences will be of great interest. The global increase in the prevalence of obesity in women [7,8] requires a better understanding of the sex-specific drivers and function of fat depots in relation to sex hormone status including menopause and post-menopause. In addition, further investigations are required to obtain a complete picture of the functions of the maternal fat depots, as the incidence of gestational diabetes mellitus and pre-eclampsia, disorders of pregnancy with short- and long-term consequences for mother and child, continues to increase due to maternal obesity [120,121]. A better understanding of the mechanisms and molecular players of sexual dimorphism will permit us to consider therapies to limit the risk of metabolic and cardiovascular pathologies in the context of central obesity and aging.

## Figures and Tables

**Table 1:** World health organization cut-off points and increased risk of metabolic complications.

Indicator	Cut-Off Points
Waist circumference (WC, cm)	>94 Men
	>80 Women
Waist-to-hip ratio (WHR)	≥0.9 Men
	≥0.85 Women
BMI (kg/m^2^)	≥30
Waist circumference (WC, cm)	>94 Men
	>80 Women
Waist-to-hip ratio (WHR)	≥0.9 Men
	≥0.85 Women
